# Dr. Wm. N. Wilson

**Published:** 1905-12

**Authors:** Frank A. Hamilton


### DR. WM. N. WILSON.

Dr. Wm. N. Wilson, of Indianapolis, formerly of Richmond, Ind., was killed October 14th by the explosion of a milk sterilizer in that city. For many years Dr. Wilson practised dentistry at Richmond, Ind. He gave up practicing dentistry and went to Indianapolis, where he engaged in the dental supply business, later becoming an instructor in the Indiana Dental College, which position he held until 1902, when he engaged in the condensed milk business. Dr. Wilson was a highly esteemed member of the Friends' Church, being a very active participant in the affairs of the church and Sunday-school, particularly in the latter. A wife, two sons and one daughter survive him. He was 52 years old.

Frank A. Hamilton.

